# Proposal for Post Hoc Quality Control in Instrumented Motion Analysis Using Markerless Motion Capture: Development and Usability Study

**DOI:** 10.2196/26825

**Published:** 2022-04-01

**Authors:** Hanna Marie Röhling, Patrik Althoff, Radina Arsenova, Daniel Drebinger, Norman Gigengack, Anna Chorschew, Daniel Kroneberg, Maria Rönnefarth, Tobias Ellermeyer, Sina Cathérine Rosenkranz, Christoph Heesen, Behnoush Behnia, Shigeki Hirano, Satoshi Kuwabara, Friedemann Paul, Alexander Ulrich Brandt, Tanja Schmitz-Hübsch

**Affiliations:** 1 Experimental and Clinical Research Center, a cooperation between the Max-Delbrück-Center for Molecular Medicine in the Helmholtz Association and the Charité - Universitätsmedizin Berlin Berlin Germany; 2 Experimental and Clinical Research Center Charité – Universitätsmedizin Berlin, corporate member of Freie Universität Berlin and Humboldt Universität zu Berlin Berlin Germany; 3 Max-Delbrück-Center for Molecular Medicine in the Helmholtz Association (MDC) Berlin Germany; 4 Motognosis GmbH Berlin Germany; 5 Department of Pediatrics St Joseph Krankenhaus Berlin-Tempelhof Berlin Germany; 6 Department of Neurology Charité – Universitätsmedizin Berlin, corporate member of Freie Universität Berlin and Humboldt Universität zu Berlin Berlin Germany; 7 Clinical Study Center Berlin Institute of Health at Charité – Universitätsmedizin Berlin Berlin Germany; 8 Department of Neurology Vivantes Auguste-Viktoria-Klinikum Berlin Germany; 9 Institute of Neuroimmunology and Multiple Sclerosis Center for Molecular Neurobiology Hamburg (ZMNH) University Medical Center Hamburg-Eppendorf Hamburg Germany; 10 Department of Neurology University Medical Center Hamburg-Eppendorf Hamburg Germany; 11 Department of Psychiatry Charité – Universitätsmedizin Berlin, corporate member of Freie Universität Berlin and Humboldt Universität zu Berlin Berlin Germany; 12 Department of Neurology Graduate School of Medicine Chiba University Chiba Japan; 13 NeuroCure Clinical Research Center Charité – Universitätsmedizin Berlin, corporate member of Freie Universität Berlin and Humboldt Universität zu Berlin Berlin Germany; 14 Department of Neurology University of California Irvine, CA United States

**Keywords:** instrumented motion analysis, markerless motion capture, visual perceptive computing, quality control, quality reporting, gait analysis

## Abstract

**Background:**

Instrumented assessment of motor symptoms has emerged as a promising extension to the clinical assessment of several movement disorders. The use of mobile and inexpensive technologies such as some markerless motion capture technologies is especially promising for large-scale application but has not transitioned into clinical routine to date. A crucial step on this path is to implement standardized, clinically applicable tools that identify and control for quality concerns.

**Objective:**

The main goal of this study comprises the development of a systematic quality control (QC) procedure for data collected with markerless motion capture technology and its experimental implementation to identify specific quality concerns and thereby rate the usability of recordings.

**Methods:**

We developed a post hoc QC pipeline that was evaluated using a large set of short motor task recordings of healthy controls (2010 recordings from 162 subjects) and people with multiple sclerosis (2682 recordings from 187 subjects). For each of these recordings, 2 raters independently applied the pipeline. They provided overall usability decisions and identified technical and performance-related quality concerns, which yielded respective proportions of their occurrence as a main result.

**Results:**

The approach developed here has proven user-friendly and applicable on a large scale. Raters’ decisions on recording usability were concordant in 71.5%-92.3% of cases, depending on the motor task. Furthermore, 39.6%-85.1% of recordings were concordantly rated as being of satisfactory quality whereas in 5.0%-26.3%, both raters agreed to discard the recording.

**Conclusions:**

We present a QC pipeline that seems feasible and useful for instant quality screening in the clinical setting. Results confirm the need of QC despite using standard test setups, testing protocols, and operator training for the employed system and by extension, for other task-based motor assessment technologies. Results of the QC process can be used to clean existing data sets, optimize quality assurance measures, as well as foster the development of automated QC approaches and therefore improve the overall reliability of kinematic data sets.

## Introduction

With technology rapidly advancing, instrumented motion analysis (IMA) has emerged as an auspicious tool to augment clinical decision-making in persons with motor impairments [1-5]. Applications range from complex gait laboratory equipment to consumer grade health apps, which quantify what a person can do in a standardized setting (motor capacity) or what a person does in everyday life (motor performance) [6]. Regarding motor capacity, marker-based optoelectronic motion analysis systems serve as the gold standard for other technologies [7,8] and are, for instance, successfully used in treatment planning for children with cerebral palsy [9]. However, their high cost and complexity of analysis comprise significant disadvantages for clinical use. Thus, technologies that are portable, affordable, and easy to use are more promising for large-scale application. Respective devices developed for clinical use include pressure-sensitive walkways, inertial sensors (“wearables”), and markerless motion capture systems based on consumer depth cameras [2,10]. In the following, the term IMA will be used for this more versatile subcategory of motion analysis systems.

Despite favorable properties, IMA has not been successfully integrated into wide clinical routine yet [11,12]. Although regulatory requirements for medical products address safety and accuracy within the context of use (eg, for application in specific diseases) [13-15], successful implementation of IMA further depends on acceptance from patients and clinicians. Thus, technical usability, interpretability of outcomes, and quantifiable clinical benefits play a major role in this development. Standardized and efficient quality control (QC) procedures, not only during initial development but also during advancement and application of a system, could facilitate this technological maturation process. We found such QC aspects to be largely understudied and underreported.

QC can be applied at three levels: preventive, ad hoc, and post hoc. Preventive QC is applied before data acquisition. Manufacturers or developing groups generate initial results on data quality and publish them in proof-of-concept studies, including small samples of healthy subjects and target groups for clinical application [7,8,16,17]. Such studies can identify major pitfalls and elaborate on correct usage of these systems. For technology that is already in use with a substantial number of researchers or clinicians, expert consensus can further yield guidelines to improve preventive QC [18]. Ad hoc QC is pertained during measurements. Depending on the system, operators can decide to discard, reinstruct, and rerecord upon observing deviations from standard operating procedures (SOPs) or receiving error messages. Lastly, post hoc QC is employed at the data analysis stage. One option in this context is univariate or multivariate outlier analysis based on the kinematic parameters [19-21]. However, these approaches are highly data-dependent, inept to uncover systematic errors or “false normal” parameter values, and do not provide information regarding underlying causes of data deviation. Additional post hoc QC measures constitute postprocessing tools and successive recalculation of kinematic parameters [22,23] as well as plausibility checks based on raw data [24-26]. To date, such processes have only been performed on comparatively small data sets.

In this study, we used data acquired with the emerging Motognosis Labs system (Motognosis GmbH) that extracts kinematic parameters from depth camera recordings. In recent years, this system was extensively used in a research context at our site and our cooperating sites [24-29] with a standardized protocol for short motor tasks specifically designed to assess motor capacities of people with multiple sclerosis (MS) [7,30]. Regarding preventive QC, previously established SOPs for system operators and patient instructions were used for all data analyzed herein. With respect to ad hoc QC, the software provides visual feedback regarding general subject positioning in the volume of acquisition and real-time tracking of the whole body as well as individual body parts. Regarding post hoc QC, we found previously employed approaches to be either insufficient, incomplete, or not feasible to reliably examine large amounts of data [19-21,24-26]. Likewise, review of IMA literature did not yield any standards or generalizable concepts. Thus, we propose an approach for systematic post hoc QC, enabling clinical users to prevent, detect, and eliminate data of inferior quality.

For the quality concerns considered here, we distinguish technical and performance issues. Technical issues comprise system-specific malfunctioning of hardware and software as well as artifacts specific to the recording technique, such as signal interference due to subjects’ clothing or the recording environment in the case of depth sensing technology. Performance issues can be considered less technology-specific and can be attributed either to the operator (eg, by providing faulty instructions) or to noncompliance of the recorded subject. If the latter is unrelated to the disease, it should lead to trial exclusion; however, impairment-related inability can be considered a feature of interest.

The main objectives of this study were to (1) build a post hoc QC pipeline that is efficient, user-friendly, and adaptable, enabling clinical users to make standardized and robust decisions concerning usability of individual recordings; (2) perform QC for a large number of recordings acquired at different study sites and thus investigate the types and frequencies of quality issues; and (3) analyze the feasibility of the approach.

## Methods

### Data Set

Our study was based on recordings of short, structured motor tasks captured with the Motognosis Labs system. This system relies on a consumer depth camera (Microsoft KinectV2, Microsoft Corporation) and visual perceptive computing. More precisely, the software development kit associated with the camera allows for the markerless tracking of 3D time series from 25 artificial anatomical landmarks for subjects located at 1.5 to 4.5 m from the camera. Custom Motognosis Labs algorithms employ these time series to extract kinematic parameters to quantify various aspects of motor capacity.

Data were pooled from 8 monocentric studies at 3 study sites that used software versions 1.1, 1.4, 2.0, or 2.1 as part of their protocols. These studies will be referred to using the following identifiers: ASD, CIS, Valkinect, VIMS, and WALKIMS-DA (conducted at Charité – Universitätsmedizin Berlin, Berlin, Germany); Ambos and Oprims (conducted at Universitätsklinikum Eppendorf, Hamburg, Germany); and Chiba (conducted at Chiba University, Chiba, Japan). These studies were approved by the respective institutional review boards and all subjects provided written informed consent. The data set comprised recordings from 187 persons with MS and 162 healthy controls. VIMS, Valkinect, and WALKIMS-DA included both groups, whereas the other studies contributed subjects from 1 group only. Descriptive statistics include information on gender, age, anthropometry, and disease severity in case of people with MS, as measured by the Expanded Disability Status Scale [31] (Table 1 and study-specific information in Table S1 in Multimedia Appendix 1).

All subjects performed the Perceptive Assessment in Multiple Sclerosis (PASS-MS) protocol or parts of it between December 2014 and April 2019. PASS-MS consists of 10 structured motor tasks: Postural Control (POCO), Postural Control with Dual Task (POCO-DUAL), Stepping in Place (SIP), Stand Up and Sit Down (SAS), Short Line Walk (SLW), Short Comfortable Speed Walk (SCSW), Short Maximum Speed Walk (SMSW), Pronator Drift Test, Finger-Nose Test, and Finger Tapping. The latter 3 tasks were excluded from this study, as evaluation algorithms were still in an explorative stage at the time, yielding premature claims regarding data quality. A description of the remaining tasks except POCO-DUAL can be found in Otte et al [7,30]. POCO-DUAL equates to POCO with the addition of a cognitive task (Serial 3’s subtraction). System operators had received in-depth training on how to use Motognosis Labs according to written SOPs. System SOPs included specifications of the setup, subject instructions, and rejection guidelines for recordings affected by performance and technical issues. According to the protocol, SAS, SLW, SCSW, and SMSW are recorded thrice consecutively, whereas POCO, POCO-DUAL, and SIP are recorded once. Deviations from SOPs occurred when single tasks or task repetitions were omitted, or operators decided to produce additional recordings (all of which should prompt an operator comment that is stored along with raw data of each recording). Such deviations explain incongruencies in the numbers of recordings per task (Table 1 and study-specific information in Table S2 in Multimedia Appendix 1), as all available recordings were included in this post hoc QC initiative.

**Table 1:** Demographic information about study subjects with missing data indicated as percentages and number of recordings per Perceptive Assessment in Multiple Sclerosis task subdivided by disease status.

Subject characteristics			All		HC^a^		PwMS^b^
**Demographics**							
	N (% female; % —^c^)	349 (51.6; 0.6)		162 (51.2; 1.2)		187 (51.9; 0)	
	Age (years), mean (SD; % —)	42.0 (12.2; 0.6)		38.3 (12.8; 1.2)		45.3 (10.8; 0)	
	Height (cm), mean (SD; % —)	173.1 (9.2; 2.6)		172.0 (9.6; 3.7)		174.1 (8.8; 1.6)	
	Weight (kg), mean (SD; % —)	72.9 (14.8; 8.0)		70.4 (14.6; 8.0)		75.0 (14.6; 8.0)	
	BMI (kg/m^2^), mean (SD; % —)	24.3 (4.1; 8.0)		23.8 (3.9; 8.0)		24.7 (4.3; 8.0)	
	EDSS^d^ median (range; % —)	N/A^e^		N/A		3.0 (0.0-6.5; 2.7)	
**# of recordings per PASS-MS^f^ task**							
	All	4692		2010		2682	
	POCO^g^	354		165		189	
	POCO-DUAL^h^	245		88		157	
	SCSW^i^	1043		489		554	
	SMSW^j^	907		361		546	
	SLW^k^	957		428		529	
	SIP^l^	291		131		160	
	SAS^m^	895		348		547	

^a^HC: healthy controls.

^b^PwMS: people with multiple sclerosis.

^c^—: not available.

^d^EDSS: Expanded Disability Status Scale.

^e^N/A: not applicable.

^f^PASS-MS: Perceptive Assessment in Multiple Sclerosis.

^g^POCO: Postural Control.

^h^POCO-DUAL: Postural Control with Dual Task.

^i^SCSW: Short Comfortable Speed Walk.

^j^SMSW: Short Maximum Speed Walk.

^k^SLW: Short Line Walk.

^l^SIP: Stepping in Place.

^m^SAS: Stand Up and Sit Down.

### QC Pipeline Development

The QC pipeline development comprised 2 key components. First, we implemented informative visualizations enabling raters to classify the quality of raw data from PASS-MS recordings and hence implicitly assess the reliability of associated kinematic parameters. Second, we developed an efficient rating strategy for large numbers of recordings.

For the creation of informative visualizations, videos from raw depth streams were generated to enable review of each recorded task. The depth information was further used to produce a condensed representation of each recording in the form of 3 images that are hereafter referred to as motion profiles. They comprise images of depth data averaged over time, over the vertical direction, and over the horizontal direction. As PASS-MS tasks are short and highly standardized, we assumed that major protocol deviations and technical issues would be easily identifiable from motion profiles. To allow for the detection of more subtle quality issues, we also illustrated characteristic signals that are used to calculate kinematic parameters with Motognosis Labs. Visualizations were generated using Python (version 3.7.3) and the matplotlib package (version 3.1.0). A stratified random sample from 15 people with MS and 14 healthy controls was used to test and update visualizations and determine the main rating criteria per task.

We then built a graphical user interface (GUI), which includes a rating window containing visualizations, an overall usability decision checkbox (keep, discard, undecided), and task-specific multiselect checkboxes containing the main rating criteria. Furthermore, on-demand viewers for depth videos and operator comments were integrated. The GUI was programmed in Python (version 3.7.3) using the tkinter package (version 8.6). We prepared detailed rating manuals as well as oral instructions (~45 minutes) to familiarize raters with the GUI. The entire data set (see Table 1) was subjected to ratings, such that each recording was investigated by 2 independent raters. In this step, 8 raters evaluated a total of 4692 recordings from 162 healthy controls and 187 people with MS. Raters comprised medical students, clinician scientists or researchers in other professions, and trained neurologists, all from Charité, Berlin. Among them, 6 raters had operated Motognosis Labs before, whereas 2 were new to the system. Moreover, 2 raters had been actively involved in the development of the QC pipeline, whereas 6 were new to any systematic QC of the data. After in-depth instructions, ratings were conducted individually by the raters at a self-selected speed.

### Statistical Analysis

Statistical analyses included the extraction of frequencies for overall usability decisions, rater concordance and discordance, and selected rating criteria. The former 2 were illustrated as confusion matrices. Furthermore, the median rating duration per recording was extracted from the GUI log files. Figures were produced with Python (version 3.7.3) using the matplotlib package (version 3.1.0).

## Results

### QC Pipeline Usage and Feasibility

**Figure 1:**
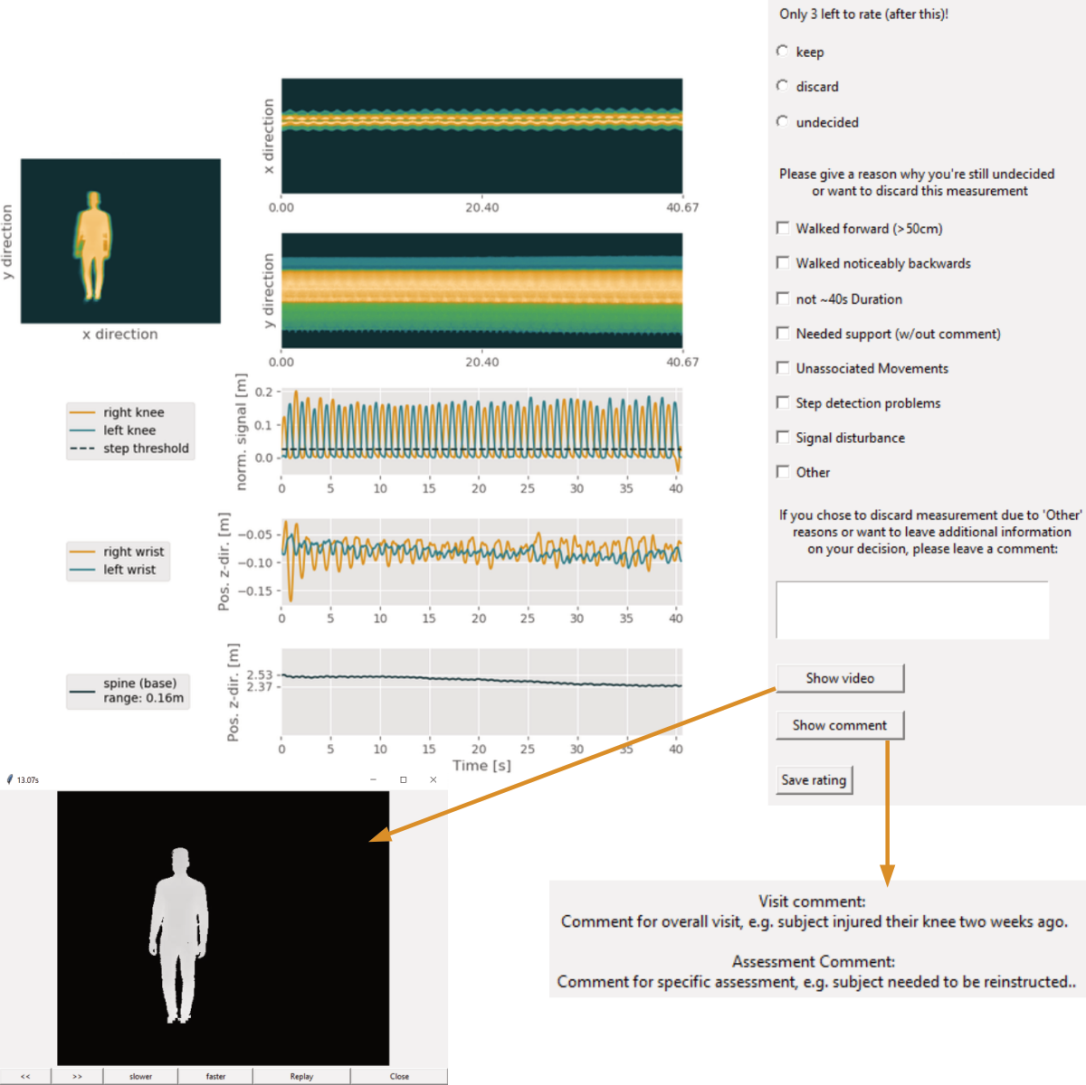
Rating window screenshots for an exemplary Stepping in Place recording. Upper left: motion profiles generated by summation of frontally recorded depth data over time, along horizontal and vertical directions and signal curves characteristic of the task (here: knee amplitudes, arm sway, and overall subject positioning over time). Upper right: checkboxes for usability decisions and main criteria including an option for free-text comments. Lower left: on-demand depth video viewer. Lower right: on-demand operator comment viewer.

After generating visualizations, the implemented GUI can be opened to progressively rate motor task recordings. Intermediate results can be saved in an underlying Excel file, such that raters can flexibly organize their workload. An example of the rating window including respective visualizations, checkboxes, and buttons is shown in Figure 1.

Oral feedback from raters upon completion confirmed that the GUI and the QC pipeline behind it were easy to use and effective. The median rating duration per recording amounted to 6.3 seconds.

### Rater Concordance and Usability of Recordings

**Figure 2:**
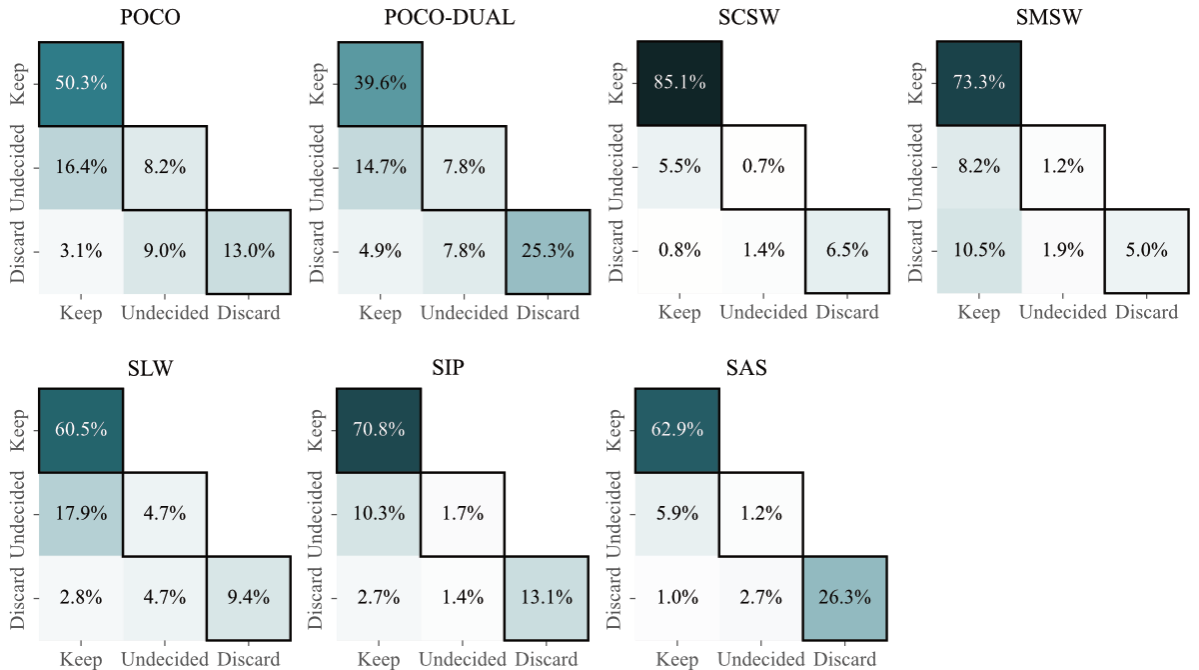
Synopsis of usability decisions by 2 raters per recording per Perceptive Assessment in Multiple Sclerosis task. Rater agreement on usability decisions keep, discard, and undecided are framed. POCO: Postural Control; POCO-DUAL: Postural Control with Dual Task; SAS: Stand Up and Sit Down; SCSW: Short Comfortable Speed Walk; SIP: Stepping in Place; SLW: short line walk; SMSW: Short Maximum Speed Walk.

Concerning keep, discard, or undecided decisions, raters concurred on more than 70% of recordings for each task (POCO: 71.5%, POCO-DUAL: 72.7%, SCSW: 92.3%, SMSW: 79.5%, SLW: 74.6%, SIP: 85.6%, and SAS: 90.4%) (Figure 2). Consequently, we observed discordance for up to 28.5% of recordings, which points to task-specific difficulties in using the rating criteria. However, such discordance was mostly due to 1 rater’s undecided decision. Instances of strictly opposing usability, meaning that 1 rater voted keep and the other discard, were uncommon (between 0.8% and 4.9%), except for SMSW (10.5%).

A task-wise visualization of rater decisions regarding usability of recordings is depicted in Figure 2. Unobjectionable usability, defined as a unanimous keep decision, was obtained for 85.1% of SCSW, more than 70% of SMSW and SIP (73.3% and 70.8%, respectively), more than 60% for SAS and SLW (62.9% and 60.5%, respectively) and less than or close to half for POCO and POCO-DUAL recordings (50.3% and 39.6%, respectively). The highest rates for unanimous discard decisions were observed for SAS (26.3%), followed by POCO-DUAL (25.3%), and POCO and SIP (13.0% and 13.1%, respectively). The respective rates were low for gait tasks including SLW, SCSW, and SMSW (9.4%, 6.5%, and 5.0%, respectively). Rater concordance as well as proportions of unanimous keep and discard decisions subdivided for all studies can be found in Table S3 in Multimedia Appendix 1.

### Main Quality Concerns

The main rating criteria compiled during QC pipeline development are listed below, with the respective tasks indicated in parentheses.

- Disturbances, technical issue: Signal disturbances including noisy background, floor, and technical issues with tracking clothing (all tasks)
- Duration, technical issue: Recording duration substantially deviating from 40 seconds, namely a deviation of more than 1 second (POCO, POCO-DUAL, and SIP)
- Step Detection, technical issue: Incorrect Step Detection (SCSW, SMSW, SIP, and SLW)
- Up/Down Phase, technical issue: Incomplete or incorrectly detected standing-up or sitting-down phase (SAS)
- Arms, performance issue: Arms not hanging loosely down at the beginning of the recording (SAS)
- Backward, performance issue: Subject walking backward by more than 50 cm or exhibiting a deliberate backward correction (SIP)
- Feet, performance issue: Deviation from closed feet position, namely if the feet are in an open or a V-shaped position (POCO and POCO-DUAL)
- Forward, performance issue: Subject moving forward by more than 50 cm (SIP)
- Movements, performance issue: Task-unassociated movements such as scratching or gesturing (POCO, POCO-DUAL, SLW, SIP, and SAS)
- Sidestep, performance issue: 1 or multiple sidesteps (POCO, POCO-DUAL, and SLW)
- Support, performance issue: Subject needing support from a walking stick, walls, rollator, or the like (all tasks)
- Other, technical or performance issue: Other/unlisted criterion (all tasks)

**Figure 3:**
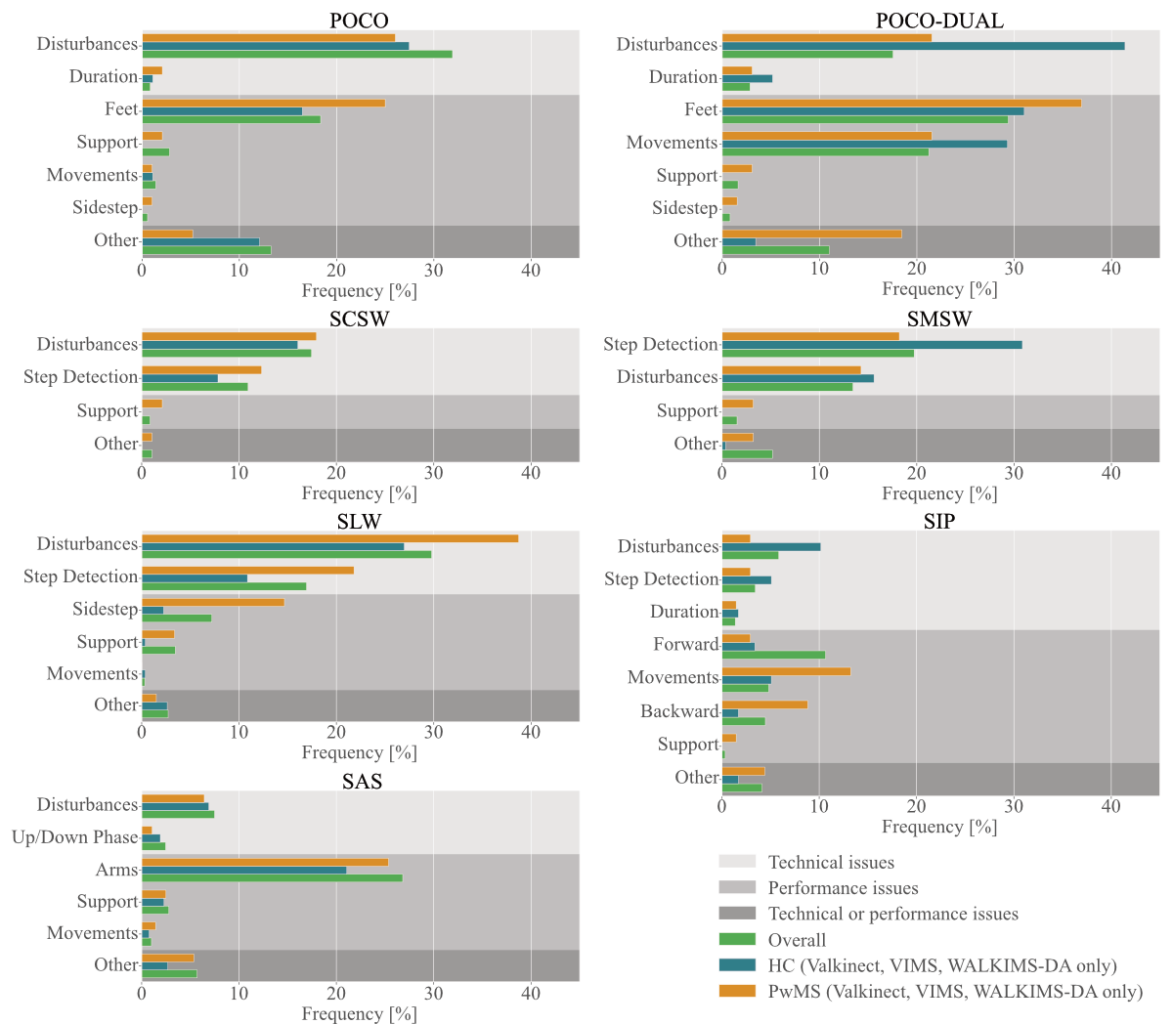
Selection frequencies of technical and performance-related rating criteria for all subjects as well as split by group for the 3 studies featuring healthy controls and people with multiple sclerosis. HC: healthy controls; POCO: Postural Control; POCO-DUAL: Postural Control with Dual Task; PwMS: people with multiple sclerosis; SAS: Stand Up and Sit Down; SCSW: Short Comfortable Speed Walk; SIP: Stepping in Place; SLW: Short Line Walk; SMSW: Short Maximum Speed Walk.

Respective selection frequencies (multiple selections were possible) are illustrated in Figure 3. Possible disease-associated differences in data quality can be estimated from the 3 studies featuring healthy controls and people with MS, namely VIMS, Valkinect, and WALKIMS-DA.

**Figure 4:**
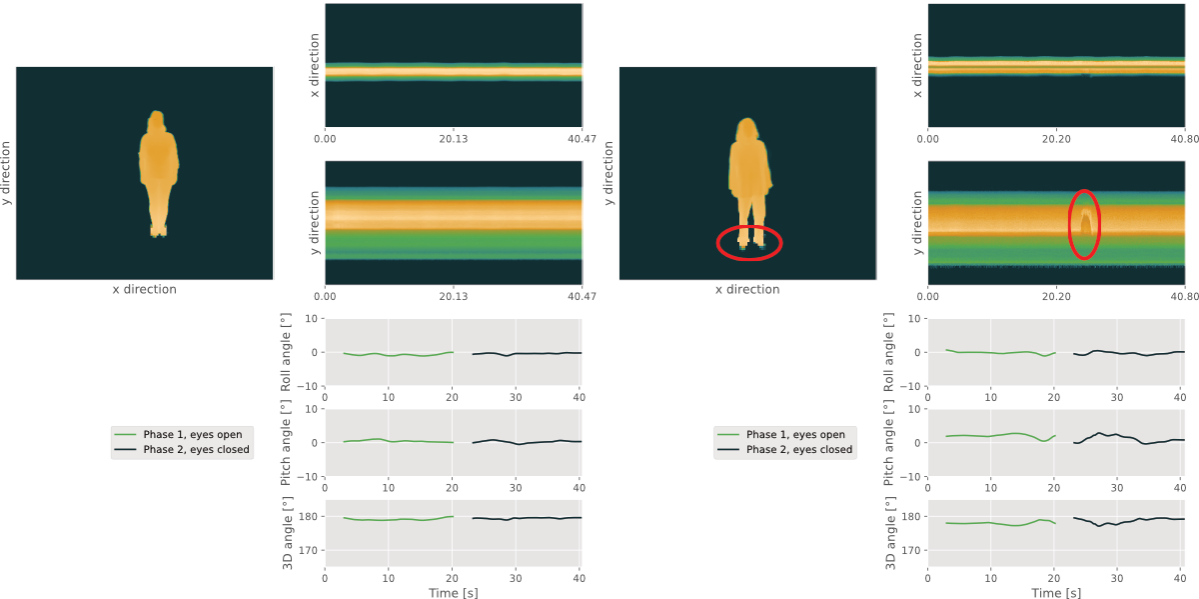
Left: quality control pipeline visualization screenshot of a high-quality Postural Control recording. Right: quality control pipeline visualization screenshot of a Postural Control recording featuring 2 frequently observed performance-related quality concerns, incorrect Feet positioning (according to standard operating procedures, the forefoot and heel should be closed) and unassociated hand Movements around second 22.

The most prevalent quality concerns comprised Feet, Disturbances, and Other for POCO and additionally Movements for POCO-DUAL. An example of a POCO recording that was discarded due to incorrect Feet positioning as well as unassociated Movements, namely the most frequent performance-associated quality concerns, can be found in Figure 4. For POCO-DUAL, supposedly task-unassociated movements were tagged with Movements and Other by the raters. However, these hand and arm movements often seemed to result from cognitive efforts made during mental arithmetic. In this case, no clear distinction between task-associated and task-unassociated movements can be made. Regarding technical quality concerns, raters’ comments suggested that recordings tagged with Disturbances or Other most often exhibited noisy or corrupt leg, feet, or floor signals.

**Figure 5:**
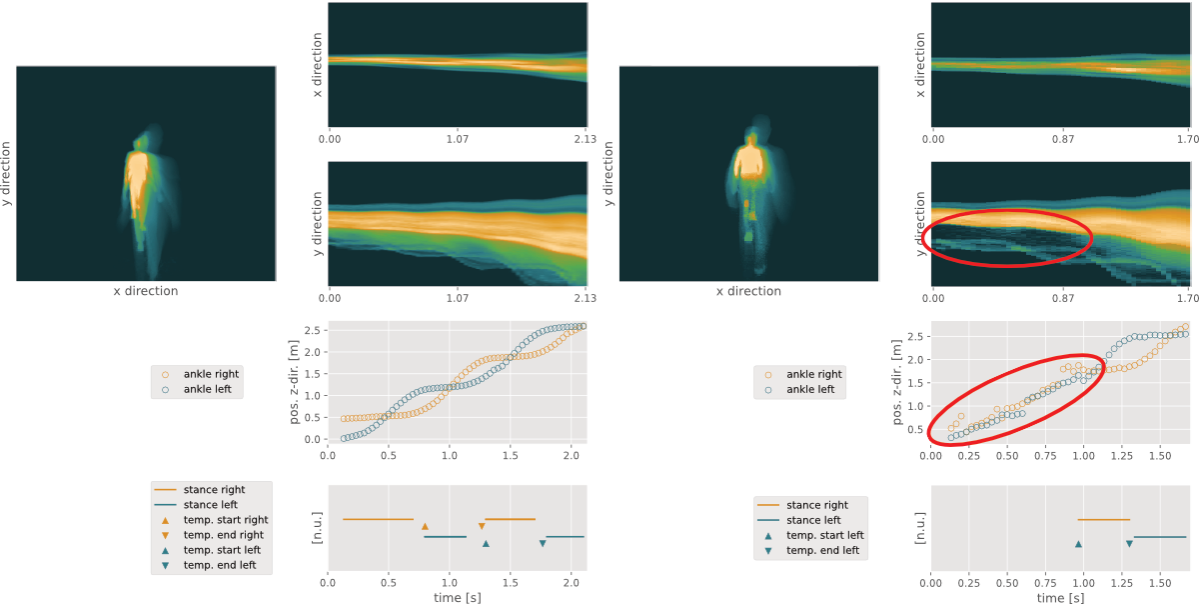
Left: quality control pipeline visualization screenshot of a high-quality short comfortable speed walk recording. Right: quality control pipeline visualization screenshot of a Short Comfortable Speed Walk recording featuring a frequently observed technical quality concern, unsuitable clothing causing Disturbances and thus Step Detection issues. Abbreviation temp. represents temporal and indicates the detected stance phases used for temporal rather than spatial parameters.

Prevalent quality concerns for gait tasks were Disturbances and Step Detection in SLW and— less frequently*—*SCSW and SMSW. A cross-dependency between the 2 criteria was often observed when unsuitable clothing led to noisy signals (noted as Disturbances by the raters), which in turn leads to issues concerning Step Detection. An example of this issue for an SCSW recording is depicted in Figure 5. Other Disturbances related to floor reflections were not associated with Step Detection issues as often.

Excessive forward locomotion (Forward) was the most frequent quality concern for SIP recordings. However, from our experience, the chosen threshold of 50 cm forward motion is rather conservative and distances up to 80-100 cm might be tolerable.

The most prominent problem for SAS was incorrect arm positioning (Arms) at the beginning of a recording. Such incorrect arm positioning was not easily discernible from the motion profile alone and raters usually consulted the provided depth videos to confirm this specific quality concern. Furthermore, a mistake in signal plot generation for SAS—affecting 3.8% of SAS plots—led to an overestimation of recordings affected by the Up/Down Phase criterion. Figure 3 provides raw ratings, and the represented numbers hence reflect this overestimation.

Disparities between people with MS and healthy controls for performance-related quality aspects were apparent for the generally less often observed Support (all tasks) and Sidestep (POCO, POCO-DUAL, and SLW) issues. This can be interpreted as a disease-related difficulty or the inability to follow task instructions. Results regarding incorrect Feet positioning during POCO and POCO-DUAL did not allow for the interpretation of this criterion as a mainly disease-related one. This criterion as well as Forward and Backward motion during SIP and the incorrect starting position of the Arms during SAS were present in both groups, though slightly more frequent in people with MS. Frequencies of the observed quality criteria further subdivided for all studies can be found in Table S4 in Multimedia Appendix 1.

## Discussion

This study presents a post hoc QC pipeline for clinical users of an IMA system. Its core consists of an interface, which enables an intuitive usability decision for individual recordings based on an extendable set of quality criteria. The pipeline proved highly feasible for users—including raters less acquainted with the IMA system itself—and yielded acceptable rater concordance. Its application in a large set of recordings from healthy controls and people with MS demonstrated the utility and necessity of post hoc QC to ensure reliable data and avoid misinterpretation of IMA results. It further identified points for improvement in preventive and ad hoc QC. To our knowledge, this is the first study to systematically investigate QC aspects and propose a clinically applicable QC pipeline for visual perceptive computing.

In the following, we will discuss 2 main aspects of our results. First, the rater concordance, which indicates the feasibility and limitations of our QC approach, and second, the usability decisions themselves, which indicate the quality and limitations of our data.

Rater concordance between 71.5% to 92.3% was generally acceptable. Only for SMSW, strictly opposed keep/discard decisions occurred to a relevant extent (10.5%). This was mostly caused by 1 rater’s discard decisions because no full gait cycle was captured. Due to the limited recording range of the depth camera, this is a frequent observation for SMSW and cannot be directly attributed to technical or performance issues. Generally, discordance may reflect ambiguity regarding rating criteria, difficulties in the evaluation of individual cases, or rater oversight. Probably only 1 rater, most likely the operator of the system, will apply post hoc QC in future clinical applications. Thus, possible reasons for rater discordance should be carefully addressed in further development of the QC pipeline, for instance, by specifying the rating criteria, as well as conducting more targeted rater trainings. However, as with other clinical judgments, QC decisions will remain informed, but ultimately intuitive decisions.

Usability decisions were interpreted as follows. Recordings receiving a unanimous keep or discard decision from the corresponding 2 raters were regarded as having assessable and satisfactory or unsatisfactory quality, respectively. Remaining recordings with discordant or undecided usability decisions were classified as needing further investigation, thus being less assessable and with potentially objectionable quality. The proportion of unanimous keep decisions varied substantially between tasks (39.6%-85.1%). In this respect, the SCSW task had the most favorable results with the highest rater concordance (92.3%) and the highest proportion of keep decisions among all tasks. At the other end of the spectrum were POCO and POCO-DUAL with rather moderate rater concordance (71.5% and 72.7%, respectively) and comparatively less unanimous keep decisions (50.3% and 39.6%, respectively). This partial ambiguity supports our inclusion of undecided as an option to avoid forced decisions as well as free text comments to enable marking of unexpected quality concerns.

Regarding technical quality issues, the short walk tasks SCSW, SMSW, and SLW suffered the most from unfavorable properties of clothing that hampered infrared light reflection [32]. POCO and POCO-DUAL often exhibited noisy and cutoff feet signals, attributable to a limited differentiation of feet and ground leading to unstable landmark estimations, as reported earlier [7]. Countermeasures include general recommendations toward subjects’ clothing and flooring at the measurement site.

We expected performance-related quality concerns to be associated with physical limitations and thus the disease status to some extent. This seemed to apply to rating criteria Sidestep and Support. However, the more commonly observed performance-related issues (eg, Feet and Movements for POCO and POCO-DUAL, Arms for SAS, and Forward for SIP) occurred in healthy subjects as well. This implies that mistakes in task instruction or ad hoc QC occurred to a relevant degree, despite detailed SOPs and operator training. Even higher proportions of performance-related issues may be expected with wider clinical use or in unsupervised telemedical applications. Thus, further IMA development should aim to implement technical measures for automated real-time detection of performance issues and respective response plans (eg, reinstruction and repetition). Performance-related quality concerns may specifically apply to the assessment of motor capacity in a lab setting or in task-based assessments as opposed to the recently proposed IMA systems for continuous assessment of motor performance [4,5,15].

In the literature, we found generally sparse reporting of QC aspects for IMA. This includes reporting of unobjectionable data quality, which we assume to be unlikely. As an indicator of technical IMA system performance, some authors reported exclusion of IMA recordings due to seemingly blatant technical failures, with rates ranging from a few corrupted examples to recordings of 48.8% of the participants [21,22,33,34]. Unfortunately, respective proportions could not be provided for our data set, as we did not track recordings discarded ad hoc. Regarding data exclusion in postprocessing, outlier detection was the most frequent approach. For univariate outlier detection on normative gait and balance parameters in children, exclusion rates of 2.5% and 6% were reported [20,35]. A multivariate outlier detection approach on kinematic gait data with successive expert evaluation identified erroneous Step Detection in 3.4% of the subjects [21], whereas a custom post hoc QC procedure applied on SMSW data obtained using Motognosis Labs led to exclusion of 6.7% of the recordings [24]. We consider the QC approach presented here to be rather conservative when compared to outlier detection. It is highly possible that significant quality concerns identified at the raw data level would not be detected by outlier analysis at the kinematic parameter level. For example, failure to stand with closed feet during POCO most likely results in reduced postural sway, which would be mistaken for higher postural stability in the respective subject at the kinematic outcome level.

Lastly, reporting of manual postprocessing, for example, using the GAITRite footfall labeling tool, is often limited to whether it was employed at all [22,36], and respective proportions are only seldom addressed [37].

Beyond IMA, the need for QC has been recognized for other technical procedures. In the context of MS research, magnetic resonance imaging and optical coherence tomography serve as examples for which recommendations have been made regarding standardized protocols, QC, and harmonious reporting thereof [38-42]. Therefore, we propose standardized reporting of IMA results to include information regarding the following: (1) number of recording failures during data acquisition; (2) type and amount of applied postprocessing, both technical and manual; (3) fraction of recordings undergoing QC; (4) fraction of recordings ultimately excluded from analysis (mention of respective causes would be highly valuable for future users)

Limitations of this study may include the decision to have each recording viewed by 2 out of 8 available raters; this limits formal interrater reliability analyses and does not assess individual rater bias. However, we did not aim to establish interrater reliability but focused on obtaining generalizable estimates of rater concordance and determining the feasibility of the approach with a reasonably diverse set of raters. Further, other possible factors influencing usability of the recordings were not specifically analyzed. These include effect of the study site, population, system operators, as well as subjects’ age, height, and weight. However, we consider QC results generalizable to and representative of routine applications because of the large size and heterogeneity of our sample. Differences in hardware were not tracked in this study (Kinect 2 sensors and laptops). Likewise, differences in software versions were disregarded because they were considered not substantial. However, recommendations regarding hardware and software may prospectively play a role in preventive QC in large-scale applications.

Regarding transferability, the visualizations employed here were specific to Motognosis Labs. However, appropriate visualizations have been implemented for other IMA systems as well. Examples include footprint depictions from pressure-sensitive walkways or acceleration illustrations from inertial sensors. Thus, we expect the general QC approach presented in this study to be transferable to other IMA systems. As for the observed quality concerns, technical issues are mostly or partially transferable to other depth camera– or visual sensor–based systems, respectively. The performance issues observed here are even more generalizable and thus highly informative for all researchers and clinicians using lab- or task-based IMA. The results of this study clearly support the need for QC of IMA data to ensure objectivity and enhance acceptance by clinical users and regulators alike. As a first step, this approach can advance consensus on the QC standards of different IMA systems and ultimately improve data quality.

## Appendix

Multimedia Appendix 1 Further information on data set, usability decisions, and rating criteria statistics.

## Abbreviations

GUI: graphical user interface
IMA: instrumented motion analysis
MS: multiple sclerosis
PASS-MS: Perceptive Assessment in Multiple Sclerosis (ie, name of short motor assessment battery recorded with Motognosis Labs)
POCO: Postural Control
POCO-DUAL: Postural Control with Dual Task
QC: quality control
SAS: Stand Up and Sit Down
SCSW: Short Comfortable Speed Walk
SIP: Stepping in Place
SLW: Short Line Walk
SMSW: Short Maximum Speed Walk
SOP: standard operating procedure

## References

[1] Petraglia F, Scarcella L, Pedrazzi G, Brancato L, Puers R, Costantino C (2019). Inertial sensors versus standard systems in gait analysis: a systematic review and meta-analysis. Eur J Phys Rehabil Med.

[2] Muro-de-la-Herran A, Garcia-Zapirain B, Mendez-Zorrilla A (2014). Gait analysis methods: an overview of wearable and non-wearable systems, highlighting clinical applications. Sensors (Basel).

[3] Pradhan C, Wuehr M, Akrami F, Neuhaeusser M, Huth S, Brandt T, Jahn K, Schniepp R (2015). Automated classification of neurological disorders of gait using spatio-temporal gait parameters. J Electromyogr Kinesiol.

[4] Frechette ML, Meyer BM, Tulipani LJ, Gurchiek RD, McGinnis RS, Sosnoff JJ (2019). Next steps in wearable technology and community ambulation in multiple sclerosis. Curr Neurol Neurosci Rep.

[5] Alexander S, Peryer G, Gray E, Barkhof F, Chataway J (2021). Wearable technologies to measure clinical outcomes in multiple sclerosis: a scoping review. Mult Scler.

[6] Maetzler W, Rochester L, Bhidayasiri R, Espay AJ, Sánchez-Ferro A, van Uem JMT (2021). Modernizing daily function assessment in Parkinson's disease using capacity, perception, and performance measures. Mov Disord.

[7] Otte K, Kayser B, Mansow-Model S, Verrel J, Paul F, Brandt AU, Schmitz-Hübsch T (2016). Accuracy and reliability of the kinect version 2 for clinical measurement of motor function. PLoS One.

[8] Webster KE, Wittwer JE, Feller JA (2005). Validity of the GAITRite® walkway system for the measurement of averaged and individual step parameters of gait. Gait Posture.

[9] Wren TAL, Tucker CA, Rethlefsen SA, Gorton GE III, Õunpuu S (2020). Clinical efficacy of instrumented gait analysis: systematic review 2020 update. Gait Posture.

[10] Morrison C, D'Souza M, Huckvale K, Dorn JF, Burggraaff J, Kamm CP, Steinheimer SM, Kontschieder P, Criminisi A, Uitdehaag B, Dahlke F, Kappos L, Sellen A (2015). Usability and acceptability of ASSESS MS: assessment of motor dysfunction in multiple sclerosis using depth-sensing computer vision. JMIR Hum Factors.

[11] Espay AJ, Hausdorff JM, Sánchez-Ferro Á, Klucken J, Merola A, Bonato P, Paul SS, Horak FB, Vizcarra JA, Mestre TA, Reilmann R, Nieuwboer A, Dorsey ER, Rochester L, Bloem BR, Maetzler W, Movement Disorder Society Task Force on Technology (2019). A roadmap for implementation of patient-centered digital outcome measures in Parkinson's disease obtained using mobile health technologies. Mov Disord.

[12] Brichetto G, Pedullà L, Podda J, Tacchino A (2019). Beyond center-based testing: understanding and improving functioning with wearable technology in MS. Mult Scler.

[13] Maetzler W, Klucken J, Horne M (2016). A clinical view on the development of technology-based tools in managing Parkinson's disease. Mov Disord.

[14] Stamate C, Magoulas GD, Kueppers S, Nomikou E, Daskalopoulos I, Jha A, Pons JS, Rothwell J, Luchini MU, Moussouri T, Iannone M, Roussos G (2018). The cloudUPDRS app: a medical device for the clinical assessment of Parkinson’s Disease. Pervasive Mob Comput.

[15] Viceconti M, Hernandez Penna S, Dartee W, Mazzà C, Caulfield B, Becker C, Maetzler W, Garcia-Aymerich J, Davico G, Rochester L (2020). Toward a regulatory qualification of real-world mobility performance biomarkers in Parkinson's patients using digital mobility outcomes. Sensors (Basel).

[16] Steinert A, Sattler I, Otte K, Röhling H, Mansow-Model S, Müller-Werdan U (2019). Using new camera-based technologies for gait analysis in older adults in comparison to the established GAITRite system. Sensors (Basel).

[17] Mancini M, Salarian A, Carlson-Kuhta P, Zampieri C, King L, Chiari L, Horak FB (2012). ISway: a sensitive, valid and reliable measure of postural control. J Neuroeng Rehabil.

[18] Kressig RW, Beauchet O, European GAITRite® Network Group (2006). Guidelines for clinical applications of spatio-temporal gait analysis in older adults. Aging Clin Exp Res.

[19] Hinton DC, Cheng Y, Paquette C (2018). Everyday multitasking habits: university students seamlessly text and walk on a split-belt treadmill. Gait Posture.

[20] Guffey K, Regier M, Mancinelli C, Pergami P (2016). Gait parameters associated with balance in healthy 2- to 4-year-old children. Gait Posture.

[21] Sunderland KM, Beaton D, Fraser J, Kwan D, McLaughlin PM, Montero-Odasso M, Peltsch AJ, Pieruccini-Faria F, Sahlas DJ, Swartz RH, Strother SC, Binns MA, ONDRI Investigators (2019). The utility of multivariate outlier detection techniques for data quality evaluation in large studies: an application within the ONDRI project. BMC Med Res Methodol.

[22] Martindale CF, Roth N, Gasner H, List J, Regensburger M, Eskofier BM, Kohl Z (2020). Technical validation of an automated mobile gait analysis system for hereditary spastic paraplegia patients. IEEE J Biomed Health Inform.

[23] Rampp A, Barth J, Schülein S, Gaßmann K-G, Klucken J, Eskofier BM (2015). Inertial sensor-based stride parameter calculation from gait sequences in geriatric patients. IEEE Trans Biomed Eng.

[24] Grobelny A, Behrens JR, Mertens S, Otte K, Mansow-Model S, Krüger T, Gusho E, Bellmann-Strobl J, Paul F, Brandt AU, Schmitz-Hübsch T (2017). Maximum walking speed in multiple sclerosis assessed with visual perceptive computing. PLoS One.

[25] Kroneberg D, Elshehabi M, Meyer A, Otte K, Doss S, Paul F, Nussbaum S, Berg D, Kühn AA, Maetzler W, Schmitz-Hübsch T (2018). Less is more–estimation of the number of strides required to assess gait variability in spatially confined settings. Front Aging Neurosci.

[26] Behrens J, Pfüller C, Mansow-Model S, Otte K, Paul F, Brandt AU (2014). Using perceptive computing in multiple sclerosis-the Short Maximum Speed Walk test. J Neuroeng Rehabil.

[27] Behrens JR, Mertens S, Krüger T, Grobelny A, Otte K, Mansow-Model S, Gusho E, Paul F, Brandt AU, Schmitz-Hübsch T (2016). Validity of visual perceptive computing for static posturography in patients with multiple sclerosis. Mult Scler.

[28] Drebinger D, Rasche L, Kroneberg D, Althoff P, Bellmann-Strobl J, Weygandt M, Paul F, Brandt AU, Schmitz-Hübsch T (2020). Association between fatigue and motor exertion in patients with multiple sclerosis—a prospective study. Front Neurol.

[29] Veauthier C, Ryczewski J, Mansow-Model S, Otte K, Kayser B, Glos M, Schöbel C, Paul F, Brandt AU, Penzel T (2019). Contactless recording of sleep apnea and periodic leg movements by nocturnal 3-D-video and subsequent visual perceptive computing. Sci Rep.

[30] Otte K, Ellermeyer T, Suzuki M, Röhling HM, Kuroiwa R, Cooper G, Mansow-Model S, Mori M, Zimmermann H, Brandt AU, Paul F, Hirano S, Kuwabara S, Schmitz-Hübsch T (2021). Cultural bias in motor function patterns: potential relevance for predictive, preventive, and personalized medicine. EPMA J.

[31] Kurtzke JF (1983). Rating neurologic impairment in multiple sclerosis: an expanded disability status scale (EDSS). Neurology.

[32] Lachat E, Macher H, Landes T, Grussenmeyer P (2015). Assessment and calibration of a RGB-D Camera (Kinect v2 Sensor) towards a potential use for close-range 3D modeling. Remote Sens.

[33] Ramsperger R, Meckler S, Heger T, van Uem J, Hucker S, Braatz U, Graessner H, Berg D, Manoli Y, Serrano JA, Ferreira JJ, Hobert MA, Maetzler W, SENSE-PARK study team (2016). Continuous leg dyskinesia assessment in Parkinson's disease—clinical validity and ecological effect. Parkinsonism Relat Disord.

[34] Larsson J, Ekvall Hansson E, Miller M (2015). Increased double support variability in elderly female fallers with vestibular asymmetry. Gait Posture.

[35] Dusing SC, Thorpe DE (2007). A normative sample of temporal and spatial gait parameters in children using the GAITRite® electronic walkway. Gait Posture.

[36] Schmitz-Hübsch T, Brandt AU, Pfueller C, Zange L, Seidel A, Kühn AA, Paul F, Minnerop M, Doss S (2016). Accuracy and repeatability of two methods of gait analysis – GaitRite™ und Mobility Lab™ – in subjects with cerebellar ataxia. Gait Posture.

[37] Wong JS, Jasani H, Poon V, Inness EL, McIlroy WE, Mansfield A (2014). Inter- and intra-rater reliability of the GAITRite system among individuals with sub-acute stroke. Gait Posture.

[38] Tewarie P, Balk L, Costello F, Green A, Martin R, Schippling S, Petzold A (2012). The OSCAR-IB consensus criteria for retinal OCT quality assessment. PLoS One.

[39] Cruz-Herranz A, Balk LJ, Oberwahrenbrock T, Saidha S, Martinez-Lapiscina EH, Lagreze WA, Schuman JS, Villoslada P, Calabresi P, Balcer L, Petzold A, Green AJ, Paul F, Brandt AU, Albrecht P, IMSVISUAL consortium (2016). The APOSTEL recommendations for reporting quantitative optical coherence tomography studies. Neurology.

[40] Traboulsee A, Simon JH, Stone L, Fisher E, Jones DE, Malhotra A, Newsome SD, Oh J, Reich DS, Richert N, Rammohan K, Khan O, Radue E-W, Ford C, Halper J, Li D (2016). Revised recommendations of the consortium of MS Centers Task Force for a standardized MRI protocol and clinical guidelines for the diagnosis and follow-up of multiple sclerosis. AJNR Am J Neuroradiol.

[41] Motamedi S, Gawlik K, Ayadi N, Zimmermann HG, Asseyer S, Bereuter C, Mikolajczak J, Paul F, Kadas EM, Brandt AU (2019). Normative data and minimally detectable change for inner retinal layer thicknesses using a semi-automated OCT image segmentation pipeline. Front Neurol.

[42] Chien C, Juenger V, Scheel M, Brandt AU, Paul F (2020). Considerations for mean upper cervical cord area implementation in a longitudinal MRI setting: methods, interrater reliability, and MRI quality control. AJNR Am J Neuroradiol.

