# Supplementary material for: Proposal for Post Hoc Quality Control in Instrumented Motion Analysis Using Markerless Motion Capture: Development and Usability Study
Source: JMIR Hum Factors. 2022 Apr 1;9(2):e26825. doi: 10.2196/26825 (PMC9015782; doi:10.2196/26825)
Supplement: Multimedia Appendix 1 [file humanfactors_v9i2e26825_app1.docx]

This is a Multimedia Appendix to a full manuscript published in JMIR Human Factors. For full copyright and citation information see <http://dx.doi.org/>10.2196/26825

Table S1: Demographic information about study subjects subdivided by study and disease status with missing data indicated as percentage. BMI: body mass index; EDSS: Expanded Disability Status Scale; HC: healthy controls; na: information not available or not applicable in case of EDSS; PwMS: people with multiple sclerosis; SD: standard deviation.

| Study | Group | N (% female; % na) | Age (years) mean (SD; % na) | Height (cm) mean (SD; % na) | Weight (kg) mean (SD; % na) | BMI (kg/m^2^) mean (SD; % na) | EDSS median (range; % na) |
| --- | --- | --- | --- | --- | --- | --- | --- |
|  |  |  |  |  |  |  |  |
| **All studies** |  |  |  |  |  |  |  |
|  | All | 349 (51.6; 0.6) | 42.0 (12.2; 0.6) | 173.1 (9.2; 2.6) | 72.9 (14.8; 8.0) | 24.3 (4.1; 8.0) |  |
|  | HC | 162 (51.2; 1.2) | 38.3 (12.8; 1.2) | 172.0 (9.6; 3.7) | 70.4 (14.6; 8.0) | 23.8 (3.9; 8.0) |  |
|  | PwMS | 187 (51.9; 0) | 45.3 (10.8; 0) | 174.1 (8.8; 1.6) | 75.0 (14.6; 8.0) | 24.7 (4.3; 8.0) | 3.0 (0.0-6.5; 2.7) |
| **Ambos** |  |  |  |  |  |  |  |
|  | PwMS (All) | 26 (46.2; 0) | 52.5 (5.4; 0) | 176.0 (9.5; 0) | 77.5 (20.1; 0) | 24.8 (5.3; 0) | 5.0 (4.0-6.5; 0) |
| **ASD** |  |  |  |  |  |  |  |
|  | HC (All) | 43 (51.2; 0) | 33.1 (8.5; 0) | 174.0 (9.6; 0) | 73.0 (16.2; 0) | 24.0 (4.5; 0) |  |
| **Chiba** |  |  |  |  |  |  |  |
|  | HC (All) | 30 (36.7; 6.7) | 30.9 (7.0; 6.7) | 167.1 (8.6; 6.7) | 60.7 (9.2; 6.7) | 21.7 (2.1; 6.7) |  |
| **CIS** |  |  |  |  |  |  |  |
|  | PwMS (All) | 41 (65.9; 0) | 37.7 (9.6; 0) | 174.8 (8.4; 0) | 70.0 (11.8; 22.0) | 23.0 (3.7; 22.0) | 1.5 (0.0-5.5; 0) |
| **Oprims** |  |  |  |  |  |  |  |
|  | PwMS (All) | 25 (36.0; 0) | 52.2 (6.7; 0) | 175.0 (9.5; 4.0) | 79.5 (11.5; 4.0) | 26.0 (3.8; 4.0) | 3.0 (0.0-5.5; 0) |
| **Valkinect** |  |  |  |  |  |  |  |
|  | All | 29 (44.8; 0) | 48.2 (15.5; 0) | 173.1 (10.1; 0) | 73.5 (13.2; 0) | 24.5 (3.9; 0) |  |
|  | HC | 22 (50.0; 0) | 48.4 (16.9; 0) | 172.2 (10.3; 0) | 70.8 (11.0; 0) | 23.8 (2.6; 0) |  |
|  | PwMS | 7 (28.6; 0) | 47.4 (10.9; 0) | 175.7 (9.7; 0) | 82.0 (16.6; 0) | 26.7 (6.3; 0) | 2.0 (1.0-4.5; 0) |
| **VIMS** |  |  |  |  |  |  |  |
|  | All | 93 (51.6; 0) | 38.6 (11.9; 0) | 172.9 (9.4; 4.3) | 71.7 (13.6; 15.1) | 24.1 (3.7; 15.1) |  |
|  | HC | 36 (52.8; 0) | 34.7 (11.0; 0) | 173.4 (10.8; 5.6) | 72.2 (16.4; 25.0) | 24.5 (4.7; 25.0) |  |
|  | PwMS | 57 (50.9; 0) | 41.0 (11.8; 0) | 172.5 (8.5; 3.5) | 71.5 (12.1; 8.8) | 23.9 (3.2; 8.8) | 2.0 (0.0-6.0; 3.5) |
| **WALKIMS-DA** |  |  |  |  |  |  |  |
|  | All | 62 (61.3; 0) | 50.0 (7.2; 0) | 172.5 (7.8; 3.2) | 76.5 (14.7; 3.2) | 25.7 (4.3; 3.2) |  |
|  | HC | 31 (64.5; 0) | 49.1 (8.7; 0) | 171.8 (7.3; 6.5) | 73.8 (13.9; 6.5) | 24.9 (3.9; 6.5) |  |
|  | PwMS | 31 (58.1; 0) | 50.8 (5.1; 0) | 173.3 (8.4; 0) | 79.1 (15.1; 0) | 26.3 (4.7; 0) | 4.0 (1.5-6.0; 9.7) |

Table S2. Number of recordings per PASS-MS task, and time period of included recordings subdivided by study. CB: Charité Berlin; CU: Chiba University; HC: healthy controls; PwMS: people with multiple sclerosis; UKE: Universitätsklinikum Eppendorf.

| Study | Site | Recordings #all (#HC; #PwMS) | | | | | | | | Time period |
| --- | --- | --- | --- | --- | --- | --- | --- | --- | --- | --- |
|  |  | All tasks | POCO | POCO-DUAL | SCSW | SMSW | SLW | SIP | SAS |  |
|  |  |  |  |  |  |  |  |  |  |  |
| **All** | CB, CU, UKE | 4692 (2010; 2682) | 354 (165; 189) | 245 (88; 157) | 1043 (489; 554) | 907 (361; 546) | 957 (428; 529) | 291 (131; 160) | 895 (348; 547) | 2014/12-2019/04 |
| **Ambos** | UKE | 383 (0; 383) | 27 (0; 27) | 26 (0; 26) | 78 (0; 78) | 74 (0; 74) | 76 (0; 76) | 26 (0; 26) | 76 (0; 76) | 2017/08-2018/12 |
| **ASD** | CB | 346 (346; 0) | 44 (44; 0) | 0 (0; 0) | 130 (130; 0) | 0 (0; 0) | 129 (129; 0) | 43 (43; 0) | 0 (0; 0) | 2017/04-2017/08 |
| **Chiba** | CU | 391 (391; 0) | 30 (30; 0) | 30 (30; 0) | 91 (91; 0) | 92 (92; 0) | 32 (32; 0) | 29 (29; 0) | 87 (87; 0) | 2018/07-2018/08 |
| **CIS** | CB | 603 (0; 603) | 41 (0; 41) | 41 (0; 41) | 120 (0; 120) | 120 (0; 120) | 120 (0; 120) | 42 (0; 42) | 119 (0; 119) | 2017/06-2019/02 |
| **Oprims** | UKE | 357 (0; 357) | 25 (0; 25) | 25 (0; 25) | 72 (0; 72) | 72 (0; 72) | 67 (0; 67) | 24 (0; 24) | 72 (0; 72) | 2017/05-2019/02 |
| **Valkinect** | CB | 429 (324; 105) | 29 (22; 7) | 29 (22; 7) | 86 (65; 21) | 86 (65; 21) | 87 (66; 21) | 29 (22; 7) | 83 (62; 21) | 2017/06-2018/08 |
| **VIMS** | CB | 1385 (546; 839) | 96 (38; 58) | 94 (36; 58) | 279 (110; 169) | 276 (111; 165) | 269 (108; 161) | 98 (37; 61) | 273 (106; 167) | 2017/06-2019/04 |
| **WALKIMS-DA** | CB | 798 (403; 395) | 62 (31; 31) | 0 (0; 0) | 187 (93; 94) | 187 (93; 94) | 177 (93; 84) | 0 (0; 0) | 185 (93; 92) | 2014/12-2015/07 |

Table S3: Statistics of unanimous *keep* and unanimous *discard* usability decisions as well as rater concordance subdivided by study and by group. All values are given as percentages. HC: healthy controls; PwMS: people with MS; RC: rater concordance rate; UK: Unanimous *keep* decision from both raters; UD: Unanimous *discard* decision from both raters.

| Task | Decision | All studies | | | ASD | Ambos | CIS | Chiba | Oprims | VIMS | | | Valkinect | | | WALKIMS-DA | | |
| --- | --- | --- | --- | --- | --- | --- | --- | --- | --- | --- | --- | --- | --- | --- | --- | --- | --- | --- |
|  |  | All | HC | PwMS | HC (All) | PwMS (All) | PwMS (All) | HC (All) | PwMS (All) | All | HC | PwMS | All | HC | PwMS | All | HC | PwMS |
|  |  |  |  |  |  |  |  |  |  |  |  |  |  |  |  |  |  |  |
| **POCO** |  |  |  |  |  |  |  |  |  |  |  |  |  |  |  |  |  |  |
|  | RC | 71.5 | 67.9 | 74.6 | 70.5 | 74.1 | 65.9 | 56.7 | 84.0 | 74.0 | 68.4 | 77.6 | 72.4 | 81.8 | 42.9 | 72.6 | 64.5 | 80.6 |
|  | UK | 50.3 | 52.7 | 48.1 | 59.1 | 29.6 | 36.6 | 43.3 | 52.0 | 46.9 | 39.5 | 51.7 | 69.0 | 77.3 | 42.9 | 61.3 | 51.6 | 71.0 |
|  | UD | 13.0 | 6.7 | 18.5 | 4.5 | 22.2 | 19.5 | 6.7 | 20.0 | 22.9 | 18.4 | 25.9 | 0.0 | 0.0 | 0.0 | 1.6 | 0.0 | 3.2 |
| **POCO-DUAL** |  |  |  |  |  |  |  |  |  |  |  |  |  |  |  |  |  |  |
|  | RC | 72.7 | 76.1 | 70.7 |  | 69.2 | 70.7 | 90.0 | 76.0 | 74.5 | 80.6 | 70.7 | 51.7 | 50.0 | 57.1 |  |  |  |
|  | UK | 39.6 | 45.5 | 36.3 |  | 38.5 | 41.5 | 90.0 | 40.0 | 28.7 | 25.0 | 31.0 | 20.7 | 18.2 | 28.6 |  |  |  |
|  | UD | 25.3 | 20.5 | 28.0 |  | 23.1 | 26.8 | 0.0 | 32.0 | 37.2 | 47.2 | 31.0 | 6.9 | 4.5 | 14.3 |  |  |  |
| **SCSW** |  |  |  |  |  |  |  |  |  |  |  |  |  |  |  |  |  |  |
|  | RC | 92.3 | 94.1 | 90.8 | 92.3 | 87.2 | 84.2 | 98.9 | 94.4 | 91.4 | 90.0 | 92.3 | 95.3 | 93.8 | 100.0 | 95.7 | 96.8 | 94.7 |
|  | UK | 85.1 | 89.4 | 81.4 | 83.8 | 73.1 | 75.0 | 98.9 | 94.4 | 83.2 | 85.5 | 81.7 | 93.0 | 92.3 | 95.2 | 86.6 | 90.3 | 83.0 |
|  | UD | 6.5 | 3.9 | 8.8 | 6.9 | 11.5 | 9.2 | 0.0 | 0.0 | 7.5 | 3.6 | 10.1 | 1.2 | 0.0 | 4.8 | 9.1 | 6.5 | 11.7 |
| **SMSW** |  |  |  |  |  |  |  |  |  |  |  |  |  |  |  |  |  |  |
|  | RC | 79.5 | 70.6 | 85.3 |  | 79.7 | 92.5 | 80.4 | 93.1 | 86.6 | 83.8 | 88.5 | 52.3 | 46.2 | 71.4 | 67.4 | 62.4 | 72.3 |
|  | UK | 73.3 | 66.2 | 78.0 |  | 75.7 | 79.2 | 73.9 | 90.3 | 81.2 | 80.2 | 81.8 | 48.8 | 41.5 | 71.4 | 61.5 | 59.1 | 63.8 |
|  | UD | 5.0 | 2.8 | 6.4 |  | 1.4 | 12.5 | 1.1 | 0.0 | 5.1 | 2.7 | 6.7 | 3.5 | 4.6 | 0.0 | 5.9 | 3.2 | 8.5 |
| **SLW** |  |  |  |  |  |  |  |  |  |  |  |  |  |  |  |  |  |  |
|  | RC | 74.6 | 79.9 | 70.3 | 87.6 | 69.7 | 81.7 | 81.2 | 76.1 | 63.9 | 65.7 | 62.7 | 79.3 | 81.8 | 71.4 | 74.6 | 83.9 | 64.3 |
|  | UK | 60.5 | 70.6 | 52.4 | 73.6 | 44.7 | 66.7 | 81.2 | 55.2 | 50.2 | 57.4 | 45.3 | 67.8 | 69.7 | 61.9 | 63.8 | 78.5 | 47.6 |
|  | UD | 9.4 | 6.5 | 11.7 | 8.5 | 21.1 | 10.0 | 0.0 | 11.9 | 8.9 | 5.6 | 11.2 | 9.2 | 10.6 | 4.8 | 6.2 | 4.3 | 8.3 |
| **SIP** |  |  |  |  |  |  |  |  |  |  |  |  |  |  |  |  |  |  |
|  | RC | 85.6 | 88.5 | 83.1 | 86.0 | 76.9 | 90.5 | 93.1 | 87.5 | 81.6 | 86.5 | 78.7 | 89.7 | 90.9 | 85.7 |  |  |  |
|  | UK | 70.8 | 77.9 | 65.0 | 79.1 | 34.6 | 85.7 | 72.4 | 50.0 | 71.4 | 78.4 | 67.2 | 82.8 | 81.8 | 85.7 |  |  |  |
|  | UD | 13.1 | 10.7 | 15.0 | 7.0 | 34.6 | 4.8 | 20.7 | 33.3 | 8.2 | 8.1 | 8.2 | 6.9 | 9.1 | 0.0 |  |  |  |
| **SAS** |  |  |  |  |  |  |  |  |  |  |  |  |  |  |  |  |  |  |
|  | RC | 90.4 | 92.5 | 89.0 |  | 76.3 | 91.6 | 93.1 | 87.5 | 92.3 | 93.4 | 91.6 | 91.6 | 88.7 | 100.0 | 91.9 | 93.5 | 90.2 |
|  | UK | 62.9 | 60.3 | 64.5 |  | 61.8 | 61.3 | 21.8 | 68.1 | 49.8 | 48.1 | 50.9 | 85.5 | 87.1 | 81.0 | 90.8 | 92.5 | 89.1 |
|  | UD | 26.3 | 31.6 | 22.9 |  | 11.8 | 30.3 | 71.3 | 12.5 | 42.1 | 45.3 | 40.1 | 3.6 | 0.0 | 14.3 | 0.5 | 0.0 | 1.1 |

Table S4: Frequencies of selected rating criteria in percent (multiple criteria could be selected for one recording) subdivided by study and by group. All values are given as percentages. HC: healthy controls; PwMS: people with MS.

| Task | Rating Criterion | Group | All studies | Ambos | ASD | Chiba | CIS | Oprims | Valkinect | VIMS | WALKIMS-DA |
| --- | --- | --- | --- | --- | --- | --- | --- | --- | --- | --- | --- |
|  |  |  |  |  |  |  |  |  |  |  |  |
| **POCO** |  |  |  |  |  |  |  |  |  |  |  |
|  | **Disturbances** |  |  |  |  |  |  |  |  |  |  |
|  |  | All | 31.9 | 37.0 | 36.4 | 36.7 | 46.3 | 28.0 | 31.0 | 24.0 | 29.0 |
|  |  | HC | 31.5 |  | 36.4 | 36.7 |  |  | 22.7 | 21.1 | 38.7 |
|  |  | PwMS | 32.3 | 37.0 |  |  | 46.3 | 28.0 | 57.1 | 25.9 | 19.4 |
|  | **Duration** |  |  |  |  |  |  |  |  |  |  |
|  |  | All | 0.8 | 0.0 | 0.0 | 0.0 | 0.0 | 0.0 | 0.0 | 1.0 | 3.2 |
|  |  | HC | 0.6 |  | 0.0 | 0.0 |  |  | 0.0 | 2.6 | 0.0 |
|  |  | PwMS | 1.1 | 0.0 |  |  | 0.0 | 0.0 | 0.0 | 0.0 | 6.5 |
|  | **Feet** |  |  |  |  |  |  |  |  |  |  |
|  |  | All | 18.4 | 25.9 | 0.0 | 0.0 | 31.7 | 24.0 | 0.0 | 39.6 | 1.6 |
|  |  | HC | 9.1 |  | 0.0 | 0.0 |  |  | 0.0 | 39.5 | 0.0 |
|  |  | PwMS | 26.5 | 25.9 |  |  | 31.7 | 24.0 | 0.0 | 39.7 | 3.2 |
|  | **Support** |  |  |  |  |  |  |  |  |  |  |
|  |  | All | 2.8 | 22.2 | 0.0 | 0.0 | 0.0 | 8.0 | 0.0 | 2.1 | 0.0 |
|  |  | HC | 0.0 |  | 0.0 | 0.0 |  |  | 0.0 | 0.0 | 0.0 |
|  |  | PwMS | 5.3 | 22.2 |  |  | 0.0 | 8.0 | 0.0 | 3.4 | 0.0 |
|  | **Movements** |  |  |  |  |  |  |  |  |  |  |
|  |  | All | 1.4 | 3.7 | 2.3 | 0.0 | 2.4 | 0.0 | 0.0 | 0.0 | 3.2 |
|  |  | HC | 1.2 |  | 2.3 | 0.0 |  |  | 0.0 | 0.0 | 3.2 |
|  |  | PwMS | 1.6 | 3.7 |  |  | 2.4 | 0.0 | 0.0 | 0.0 | 3.2 |
|  | **Sidestep** |  |  |  |  |  |  |  |  |  |  |
|  |  | All | 0.6 | 3.7 | 0.0 | 0.0 | 0.0 | 0.0 | 0.0 | 0.0 | 1.6 |
|  |  | HC | 0.0 |  | 0.0 | 0.0 |  |  | 0.0 | 0.0 | 0.0 |
|  |  | PwMS | 1.1 | 3.7 |  |  | 0.0 | 0.0 | 0.0 | 0.0 | 3.2 |
|  | **Other** |  |  |  |  |  |  |  |  |  |  |
|  |  | All | 13.3 | 14.8 | 20.5 | 40.0 | 12.2 | 4.0 | 6.9 | 3.1 | 17.7 |
|  |  | HC | 19.4 |  | 20.5 | 40.0 |  |  | 9.1 | 5.3 | 22.6 |
|  |  | PwMS | 7.9 | 14.8 |  |  | 12.2 | 4.0 | 0.0 | 1.7 | 12.9 |
| **POCO-DUAL** |  |  |  |  |  |  |  |  |  |  |  |
|  | **Disturbances** |  |  |  |  |  |  |  |  |  |  |
|  |  | All | 17.6 | 3.8 |  | 10.0 | 0.0 | 4.0 | 69.0 | 19.1 |  |
|  |  | HC | 30.7 |  |  | 10.0 |  |  | 72.7 | 22.2 |  |
|  |  | PwMS | 10.2 | 3.8 |  |  | 0.0 | 4.0 | 57.1 | 17.2 |  |
|  | **Duration** |  |  |  |  |  |  |  |  |  |  |
|  |  | All | 2.9 | 3.8 |  | 0.0 | 0.0 | 4.0 | 0.0 | 5.3 |  |
|  |  | HC | 3.4 |  |  | 0.0 |  |  | 0.0 | 8.3 |  |
|  |  | PwMS | 2.5 | 3.8 |  |  | 0.0 | 4.0 | 0.0 | 3.4 |  |
|  | **Feet** |  |  |  |  |  |  |  |  |  |  |
|  |  | All | 29.4 | 34.6 |  | 0.0 | 31.7 | 32.0 | 0.0 | 44.7 |  |
|  |  | HC | 20.5 |  |  | 0.0 |  |  | 0.0 | 50.0 |  |
|  |  | PwMS | 34.4 | 34.6 |  |  | 31.7 | 32.0 | 0.0 | 41.4 |  |
|  | **Movements** |  |  |  |  |  |  |  |  |  |  |
|  |  | All | 21.2 | 26.9 |  | 0.0 | 24.4 | 16.0 | 37.9 | 21.3 |  |
|  |  | HC | 19.3 |  |  | 0.0 |  |  | 40.9 | 22.2 |  |
|  |  | PwMS | 22.3 | 26.9 |  |  | 24.4 | 16.0 | 28.6 | 20.7 |  |
|  | **Support** |  |  |  |  |  |  |  |  |  |  |
|  |  | All | 1.6 | 7.7 |  | 0.0 | 0.0 | 0.0 | 0.0 | 2.1 |  |
|  |  | HC | 0.0 |  |  | 0.0 |  |  | 0.0 | 0.0 |  |
|  |  | PwMS | 2.5 | 7.7 |  |  | 0.0 | 0.0 | 0.0 | 3.4 |  |
|  | **Sidestep** |  |  |  |  |  |  |  |  |  |  |
|  |  | All | 0.8 | 0.0 |  | 0.0 | 0.0 | 4.0 | 0.0 | 1.1 |  |
|  |  | HC | 0.0 |  |  | 0.0 |  |  | 0.0 | 0.0 |  |
|  |  | PwMS | 1.3 | 0.0 |  |  | 0.0 | 4.0 | 0.0 | 1.7 |  |
|  | **Other** |  |  |  |  |  |  |  |  |  |  |
|  |  | All | 11.0 | 26.9 |  | 0.0 | 9.8 | 8.0 | 0.0 | 14.9 |  |
|  |  | HC | 2.3 |  |  | 0.0 |  |  | 0.0 | 5.6 |  |
|  |  | PwMS | 15.9 | 26.9 |  |  | 9.8 | 8.0 | 0.0 | 20.7 |  |
| **SCSW** |  |  |  |  |  |  |  |  |  |  |  |
|  | **Disturbances** |  |  |  |  |  |  |  |  |  |  |
|  |  | All | 17.4 | 16.7 | 12.3 | 4.4 | 41.7 | 6.9 | 8.1 | 21.5 | 14.4 |
|  |  | HC | 12.9 |  | 12.3 | 4.4 |  |  | 9.2 | 27.3 | 7.5 |
|  |  | PwMS | 21.5 | 16.7 |  |  | 41.7 | 6.9 | 4.8 | 17.8 | 21.3 |
|  | **Step Detection** |  |  |  |  |  |  |  |  |  |  |
|  |  | All | 10.9 | 24.4 | 11.5 | 1.1 | 15.8 | 5.6 | 3.5 | 10.8 | 12.3 |
|  |  | HC | 7.6 |  | 11.5 | 1.1 |  |  | 3.1 | 9.1 | 9.7 |
|  |  | PwMS | 13.9 | 24.4 |  |  | 15.8 | 5.6 | 4.8 | 11.8 | 14.9 |
|  | **Support** |  |  |  |  |  |  |  |  |  |  |
|  |  | All | 0.9 | 3.8 | 0.0 | 0.0 | 0.0 | 0.0 | 0.0 | 1.1 | 1.6 |
|  |  | HC | 0.0 |  | 0.0 | 0.0 |  |  | 0.0 | 0.0 | 0.0 |
|  |  | PwMS | 1.6 | 3.8 |  |  | 0.0 | 0.0 | 0.0 | 1.8 | 3.2 |
|  | **Other** |  |  |  |  |  |  |  |  |  |  |
|  |  | All | 1.1 | 0.0 | 3.8 | 0.0 | 2.5 | 0.0 | 0.0 | 0.7 | 0.5 |
|  |  | HC | 1.0 |  | 3.8 | 0.0 |  |  | 0.0 | 0.0 | 0.0 |
|  |  | PwMS | 1.1 | 0.0 |  |  | 2.5 | 0.0 | 0.0 | 1.2 | 1.1 |
| **SMSW** |  |  |  |  |  |  |  |  |  |  |  |
|  | **Step Detection** |  |  |  |  |  |  |  |  |  |  |
|  |  | All | 19.7 | 14.9 |  | 7.6 | 19.2 | 5.6 | 51.2 | 7.6 | 36.9 |
|  |  | HC | 24.9 |  |  | 7.6 |  |  | 58.5 | 7.2 | 39.8 |
|  |  | PwMS | 16.3 | 14.9 |  |  | 19.2 | 5.6 | 28.6 | 7.9 | 34.0 |
|  | **Disturbances** |  |  |  |  |  |  |  |  |  |  |
|  |  | All | 13.5 | 13.5 |  | 4.3 | 16.7 | 8.3 | 11.6 | 19.6 | 9.6 |
|  |  | HC | 12.7 |  |  | 4.3 |  |  | 12.3 | 26.1 | 5.4 |
|  |  | PwMS | 13.9 | 13.5 |  |  | 16.7 | 8.3 | 9.5 | 15.2 | 13.8 |
|  | **Support** |  |  |  |  |  |  |  |  |  |  |
|  |  | All | 1.5 | 6.8 |  | 0.0 | 0.0 | 0.0 | 0.0 | 2.2 | 1.6 |
|  |  | HC | 0.0 |  |  | 0.0 |  |  | 0.0 | 0.0 | 0.0 |
|  |  | PwMS | 2.6 | 6.8 |  |  | 0.0 | 0.0 | 0.0 | 3.6 | 3.2 |
|  | **Other** |  |  |  |  |  |  |  |  |  |  |
|  |  | All | 5.2 | 10.8 |  | 22.8 | 5.8 | 1.4 | 0.0 | 2.5 | 1.6 |
|  |  | HC | 6.1 |  |  | 22.8 |  |  | 0.0 | 0.9 | 0.0 |
|  |  | PwMS | 4.6 | 10.8 |  |  | 5.8 | 1.4 | 0.0 | 3.6 | 3.2 |
| **SLW** |  |  |  |  |  |  |  |  |  |  |  |
|  | **Disturbances** |  |  |  |  |  |  |  |  |  |  |
|  |  | All | 29.8 | 25.0 | 24.8 | 9.4 | 34.2 | 22.4 | 20.7 | 42.0 | 24.9 |
|  |  | HC | 25.0 |  | 24.8 | 9.4 |  |  | 22.7 | 39.8 | 15.1 |
|  |  | PwMS | 33.6 | 25.0 |  |  | 34.2 | 22.4 | 14.3 | 43.5 | 35.7 |
|  | **Step Detection** |  |  |  |  |  |  |  |  |  |  |
|  |  | All | 16.9 | 15.8 | 18.6 | 9.4 | 18.3 | 20.9 | 20.7 | 13.4 | 18.6 |
|  |  | HC | 13.1 |  | 18.6 | 9.4 |  |  | 15.2 | 11.1 | 7.5 |
|  |  | PwMS | 20.0 | 15.8 |  |  | 18.3 | 20.9 | 38.1 | 14.9 | 31.0 |
|  | **Sidestep** |  |  |  |  |  |  |  |  |  |  |
|  |  | All | 7.2 | 11.8 | 1.6 | 0.0 | 2.5 | 14.9 | 1.1 | 8.9 | 11.3 |
|  |  | HC | 1.9 |  | 1.6 | 0.0 |  |  | 1.5 | 2.8 | 2.2 |
|  |  | PwMS | 11.5 | 11.8 |  |  | 2.5 | 14.9 | 0.0 | 13.0 | 21.4 |
|  | **Support** |  |  |  |  |  |  |  |  |  |  |
|  |  | All | 3.4 | 19.7 | 0.0 | 0.0 | 0.8 | 10.4 | 0.0 | 3.7 | 0.0 |
|  |  | HC | 0.2 |  | 0.0 | 0.0 |  |  | 0.0 | 0.9 | 0.0 |
|  |  | PwMS | 6.0 | 19.7 |  |  | 0.8 | 10.4 | 0.0 | 5.6 | 0.0 |
|  | **Movements** |  |  |  |  |  |  |  |  |  |  |
|  |  | All | 0.3 | 1.3 | 0.8 | 0.0 | 0.0 | 0.0 | 0.0 | 0.4 | 0.0 |
|  |  | HC | 0.5 |  | 0.8 | 0.0 |  |  | 0.0 | 0.9 | 0.0 |
|  |  | PwMS | 0.2 | 1.3 |  |  | 0.0 | 0.0 | 0.0 | 0.0 | 0.0 |
|  | **Other** |  |  |  |  |  |  |  |  |  |  |
|  |  | All | 2.7 | 6.6 | 0.0 | 12.5 | 2.5 | 4.5 | 2.3 | 0.7 | 4.0 |
|  |  | HC | 2.6 |  | 0.0 | 12.5 |  |  | 1.5 | 0.9 | 5.4 |
|  |  | PwMS | 2.8 | 6.6 |  |  | 2.5 | 4.5 | 4.8 | 0.6 | 2.4 |
| **SAS** |  |  |  |  |  |  |  |  |  |  |  |
|  | **Disturbances** |  |  |  |  |  |  |  |  |  |  |
|  |  | All | 7.5 | 6.6 |  | 0.0 | 11.8 | 16.7 | 9.6 | 4.8 | 8.1 |
|  |  | HC | 5.2 |  |  | 0.0 |  |  | 12.9 | 2.8 | 7.5 |
|  |  | PwMS | 9.0 | 6.6 |  |  | 11.8 | 16.7 | 0.0 | 6.0 | 8.7 |
|  | **Up/Down Phase** |  |  |  |  |  |  |  |  |  |  |
|  |  | All | 2.5 | 3.9 |  | 4.6 | 4.2 | 2.8 | 0.0 | 2.9 | 0.0 |
|  |  | HC | 2.6 |  |  | 4.6 |  |  | 0.0 | 4.7 | 0.0 |
|  |  | PwMS | 2.4 | 3.9 |  |  | 4.2 | 2.8 | 0.0 | 1.8 | 0.0 |
|  | **Arms** |  |  |  |  |  |  |  |  |  |  |
|  |  | All | 26.8 | 3.9 |  | 74.7 | 31.9 | 11.1 | 4.8 | 43.6 | 1.6 |
|  |  | HC | 34.5 |  |  | 74.7 |  |  | 1.6 | 48.1 | 3.2 |
|  |  | PwMS | 21.9 | 3.9 |  |  | 31.9 | 11.1 | 14.3 | 40.7 | 0.0 |
|  | **Support** |  |  |  |  |  |  |  |  |  |  |
|  |  | All | 2.8 | 14.5 |  | 0.0 | 0.8 | 0.0 | 0.0 | 2.9 | 2.7 |
|  |  | HC | 1.7 |  |  | 0.0 |  |  | 0.0 | 4.7 | 1.1 |
|  |  | PwMS | 3.5 | 14.5 |  |  | 0.8 | 0.0 | 0.0 | 1.8 | 4.3 |
|  | **Movements** |  |  |  |  |  |  |  |  |  |  |
|  |  | All | 1.0 | 0.0 |  | 0.0 | 1.7 | 1.4 | 1.2 | 1.8 | 0.0 |
|  |  | HC | 0.6 |  |  | 0.0 |  |  | 0.0 | 1.9 | 0.0 |
|  |  | PwMS | 1.3 | 0.0 |  |  | 1.7 | 1.4 | 4.8 | 1.8 | 0.0 |
|  | **Other** |  |  |  |  |  |  |  |  |  |  |
|  |  | All | 5.7 | 18.4 |  | 4.6 | 3.4 | 9.7 | 3.6 | 5.1 | 2.7 |
|  |  | HC | 3.2 |  |  | 4.6 |  |  | 3.2 | 2.8 | 2.2 |
|  |  | PwMS | 7.3 | 18.4 |  |  | 3.4 | 9.7 | 4.8 | 6.6 | 3.3 |
| **SIP** |  |  |  |  |  |  |  |  |  |  |  |
|  | **Disturbances** |  |  |  |  |  |  |  |  |  |  |
|  |  | All | 5.8 | 7.7 | 7.0 | 10.3 | 2.4 | 0.0 | 6.9 | 6.1 |  |
|  |  | HC | 9.2 |  | 7.0 | 10.3 |  |  | 9.1 | 10.8 |  |
|  |  | PwMS | 3.1 | 7.7 |  |  | 2.4 | 0.0 | 0.0 | 3.3 |  |
|  | **Step Detection** |  |  |  |  |  |  |  |  |  |  |
|  |  | All | 3.4 | 19.2 | 0.0 | 0.0 | 0.0 | 0.0 | 6.9 | 3.1 |  |
|  |  | HC | 2.3 |  | 0.0 | 0.0 |  |  | 4.5 | 5.4 |  |
|  |  | PwMS | 4.4 | 19.2 |  |  | 0.0 | 0.0 | 14.3 | 1.6 |  |
|  | **Duration** |  |  |  |  |  |  |  |  |  |  |
|  |  | All | 1.4 | 0.0 | 4.7 | 0.0 | 0.0 | 0.0 | 0.0 | 2.0 |  |
|  |  | HC | 2.3 |  | 4.7 | 0.0 |  |  | 0.0 | 2.7 |  |
|  |  | PwMS | 0.6 | 0.0 |  |  | 0.0 | 0.0 | 0.0 | 1.6 |  |
|  | **Forward** |  |  |  |  |  |  |  |  |  |  |
|  |  | All | 10.7 | 34.6 | 11.6 | 17.2 | 2.4 | 29.2 | 3.4 | 3.1 |  |
|  |  | HC | 9.2 |  | 11.6 | 17.2 |  |  | 4.5 | 2.7 |  |
|  |  | PwMS | 11.9 | 34.6 |  |  | 2.4 | 29.2 | 0.0 | 3.3 |  |
|  | **Movements** |  |  |  |  |  |  |  |  |  |  |
|  |  | All | 4.8 | 3.8 | 0.0 | 0.0 | 2.4 | 0.0 | 0.0 | 12.2 |  |
|  |  | HC | 2.3 |  | 0.0 | 0.0 |  |  | 0.0 | 8.1 |  |
|  |  | PwMS | 6.9 | 3.8 |  |  | 2.4 | 0.0 | 0.0 | 14.8 |  |
|  | **Backward** |  |  |  |  |  |  |  |  |  |  |
|  |  | All | 4.5 | 0.0 | 0.0 | 0.0 | 2.4 | 20.8 | 3.4 | 6.1 |  |
|  |  | HC | 0.8 |  | 0.0 | 0.0 |  |  | 4.5 | 0.0 |  |
|  |  | PwMS | 7.5 | 0.0 |  |  | 2.4 | 20.8 | 0.0 | 9.8 |  |
|  | **Support** |  |  |  |  |  |  |  |  |  |  |
|  |  | All | 0.3 | 0.0 | 0.0 | 0.0 | 0.0 | 0.0 | 0.0 | 1.0 |  |
|  |  | HC | 0.0 |  | 0.0 | 0.0 |  |  | 0.0 | 0.0 |  |
|  |  | PwMS | 0.6 | 0.0 |  |  | 0.0 | 0.0 | 0.0 | 1.6 |  |
|  | **Other** |  |  |  |  |  |  |  |  |  |  |
|  |  | All | 4.1 | 11.5 | 0.0 | 3.4 | 4.8 | 8.3 | 0.0 | 4.1 |  |
|  |  | HC | 1.5 |  | 0.0 | 3.4 |  |  | 0.0 | 2.7 |  |
|  |  | PwMS | 6.2 | 11.5 |  |  | 4.8 | 8.3 | 0.0 | 4.9 |  |
